# A multi-tissue de novo transcriptome assembly and relative gene expression of the vulnerable freshwater salmonid *Thymallus ligericus*

**DOI:** 10.1007/s10709-024-00210-7

**Published:** 2024-06-18

**Authors:** Giulia Secci-Petretto, Steven Weiss, André Gomes-dos-Santos, Henri Persat, André M. Machado, Inês Vasconcelos, L. Filipe C. Castro, Elsa Froufe

**Affiliations:** 1 CIIMAR/CIMAR, Interdisciplinary Centre of Marine and Environmental Research, University of Porto, Terminal de Cruzeiros do Porto de Leixões, Avenida General Norton de Matos, S/N, 4450-208 Matosinhos, Portugal; 2 Department of Biology, Faculty of Sciences, U. Porto - University of Porto, Porto, Portugal; 3 Institute of Biology, University of Graz, Universitätsplatz 2, 8010 Graz, Austria; 4 Société Française d’Ichthyologie, Muséum National d’Histoire Naturelle Paris, France, 57 Rue Cuvier CP26, 75005 Paris, France

**Keywords:** *Thymallus*, Transcriptome assembly, Relative gene expression, Salmonidae

## Abstract

Freshwater ecosystems are among the most endangered ecosystems worldwide. While numerous taxa are on the verge of extinction as a result of global changes and direct or indirect anthropogenic activity, genomic and transcriptomic resources represent a key tool for comprehending species' adaptability and serve as the foundation for conservation initiatives. The Loire grayling, *Thymallus ligericus*, is a freshwater European salmonid endemic to the upper Loire River basin. The species is comprised of fragmented populations that are dispersed over a small area and it has been identified as a vulnerable species. Here, we provide a multi-tissue de novo transcriptome assembly of *T. ligericus*. The completeness and integrity of the transcriptome were assessed before and after redundancy removal with lineage-specific libraries from Eukaryota, Metazoa, Vertebrata, and Actinopterygii. Relative gene expression was assessed for each of the analyzed tissues, using the de novo assembled transcriptome and a genome-based analysis using the available *T. thymallus* genome as a reference. The final assembly, with a contig N50 of 1221 and Benchmarking Universal Single-Copy Orthologs (BUSCO) scores above 94%, is made accessible along with structural and functional annotations and relative gene expression of the five tissues (NCBI SRA and FigShare databases). This is the first transcriptomic resource for this species, which provides a foundation for future research on this and other salmonid species that are increasingly exposed to environmental stressors.

## Introduction

Freshwater fishes are among the most threatened vertebrates. In Europe for example, they are the second most threatened group of organisms following molluscs (Costa et al. 2021). Of the 434 fish species reported in the IUCN red list, an estimated 41.2% are threatened, salmonids being the second most affected family (Costa et al. 2021). The most common threats affecting freshwater fauna in Europe include habitat fragmentation, pollution, the presence of invasive species, dams and hydropower plants and climate change (Mueller et al. 2020). Thymallinae (graylings), a monogeneric subfamily in the diverse Salmonidae family, is represented solely by the genus *Thymallus*, commonly named grayling*.* According to the latest revision, 15 taxa are currently listed in the genus (Weiss et al. 2021). The highest diversity occurs in the Asian continent, where 12 of the 15 known species are found (Weiss et al. 2021). One of these, *Thymallus arcticus* (Pallas 1776), also inhabits some parts of North America, more specifically Alaska, Montana and Canada (Weiss et al. 2021). Three species are recognized in Europe: the widely distributed European grayling *Thymallus thymallus* (Linnaeus, 1758), the Adriatic grayling *Thymallus aeliani* Valenciennes, 1848 and the Loire grayling *Thymallus ligericus* Persat et al., 2019. Until a few years ago, the latter two were considered different lineages of *T. thymallus*. *Thymallus ligericus* is the most recently described *Thymallus* species (Persat et al. 2019) and a revision of the genus categorized it as vulnerable, based on its distribution range and the documented habitat fragmentation (Persat et al. 2016; Weiss et al. 2021). This grayling inhabits the upper reaches of the Loire River (France) and some of its main tributaries, such as the Vienne, the Allier, the Sioule and the Lignon Rivers (Persat et al. 2016; Weiss et al. 2021). Populations of *T. ligericus* have maintained their reproductive isolation despite decades of failed restocking attempts as demonstrated by the *T. ligericus* population in the Vienne River, which has been confronted with hatchery-reared *T. thymallus* over 50 years, yet no trace of these stocked individuals can be found (Persat et al. 2016).

The impact of environmental changes on species functions such as physiology, behaviour, and gene expression, is relatively unexplored in Thymallinae. Studies exist for the widely distributed *T. thymallus* and *T. arcticus*, as well as for other salmonid species and can offer insights into how environmental stressors may affect *T. ligericus* and other Thymallinae. Indeed it is demonstrated that events such as rising temperature, discharge fluctuations and human pollution affect physiological functions in *T. thymallus* and *T. arcticus*, as well as *Salmo trutta* Linnaeus, 1758 *and Salmo salar* Linnaeus, 1758 e.g. by inducing male bias, disrupting reproduction, growth, recruitment and feeding and swimming behaviours (Newcombe and Jensen 1996; Deegan et al. 1999; Luecke and MacKinnon 2008; Wedekind et al. 2013; Richard et al. 2015; Warren et al. 2015; Bašić et al. 2018; Opinion et al. 2020; Hayes et al. 2021; Auer et al. 2022). Thymallinae are strictly freshwater salmonids and their migratory behaviour within the same watercourse during the spawning season (West et al. 1992; Hughes and Reynolds 1994; Meyer 2001; Heim et al. 2015; Zuev et al. 2021) make them particularly sensitive to stream disturbances. The Loire is one of Europe’s longest rivers. Its first largest tributary, the Allier River, includes several biodiversity hotspots for plants and birds (Moatar et al. 2022) and hosts populations of *T. ligericus*. Some major urbanization of the Loire basin has arisen in the Allier Valley, impacting the environment with anthropogenic activities such as the construction of dams (Moatar et al. 2022). In 1994 the implementation of the “Loire Grandeur Nature Plan” gradually began to restore and improve the management of the Loire and Allier River environments, which had suffered from unstopped anthropogenic activities for decades (Moatar et al. 2022). Despite this, the effects of climate change have been visible in this area and the Allier River, an important regional resource in terms of domestic and irrigation water supply, has recently experienced extreme dry summers with insufficient winter recharge (Labbe et al. 2023). These extreme conditions have inevitable consequences on the ecosystem’s biota and developing mechanisms to cope with such environmental fluctuations is critical for organisms’ survival. Understanding the limits of species resilience is vital to ensure the preservation and correct management of ecosystems. Genomic and transcriptomic resources, coupled with studies of ecology and physiology, are key tools that can help uncover compensatory mechanisms such as gene expression regulation in extreme environmental conditions. Increasingly lower sequencing costs coupled with advances in high-throughput sequencing techniques allow the generation of genomic and transcriptomic resources for non-model species (Connon et al. 2018; Gomes-dos-Santos et al. 2022). Among Thymallinae, transcriptomic resources have only been generated for *T. thymallus* (Pasquier et al. 2016; Varadharajan et al. 2018), which is also the only species of the genus with an available whole genome assembly (Varadharajan et al. 2018; Savilammi et al. 2019). Given the critical conservation status of *T. ligericus,* the lack of transcriptomic resources for this species may hinder future efforts to prevent its decline. In this work, we generate a high-quality multi-tissue transcriptome of the vulnerable species *Thymallus ligericus* from the Alagnon River (Allier basin). By pooling information from five different tissues, we aim to ensure the robustness of the resulting transcriptome assembly. Additionally, we provide relative gene expression profiles of the five tissues. As no transcriptomic resources are yet available for this taxon, this work provides a valuable framework for future studies that will assess the species' ability to respond to stressors such as climate change and anthropogenic activity, to which *T. ligericus* is constantly exposed.

## Methods

### Animal sampling

**Fig. 1:**
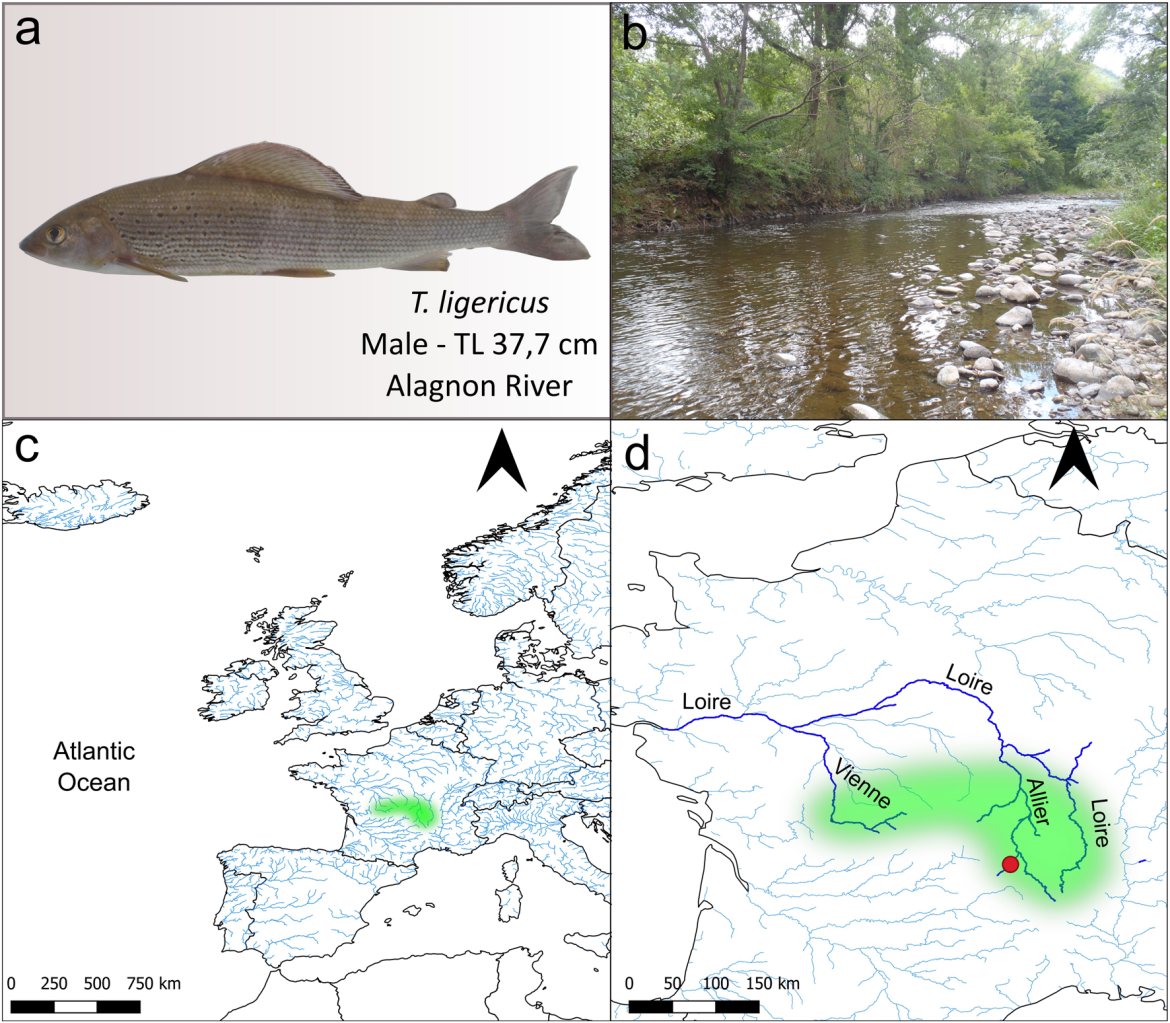
Specimen analyzed and detailed representation of the species’ area of occurrence. **a** Male *T. ligericus* analyzed in the present work. TL: total length. **b** Photo of the Alagnon River section where the specimen was collected. **c** Map with approximate distribution (marked in green) of *T. ligericus* based on available literature (Persat et al. 2016; Weiss et al. 2021). **d** Zoom in on the species distribution in the Upper Loire River. The red dot identifies the sampling point along the Alagnon River where the specimen was caught

At the end of the summer season (27/08/2022), one adult male of *T. ligericus* was caught by angling from the Alagnon River (45° 14′ 34.4 N-3° 10′ 23″ E; Fig. 1) in the Massiac district (Loire, France). Afterwards, 1cm^3^ of tissue was sampled from the brain, gills, gonad, liver, and muscle. Tissues were then stored in RNA-later (Invitrogen, Thermo Fisher Scientific) at 4 °C for 24 h and later at − 80 °C in preparation for RNA extraction. The samples were collected with the permission of the Direction Départementale des Territoires-Préfète de la Loire and genetic analyses were carried out according to the Nagoya Protocol (reference permit number: TREL2206915S/602).

### RNA extraction, sequencing and library construction

Total RNA was extracted from the five tissues using the NZY Total RNA isolation kit (NZYtech) following the manufacturer´s instructions. RNA concentration was measured with DeNovix DS-11 FS and its integrity was assessed by electrophoresis in 1% agarose gel. The five samples were then sent in dry ice to Macrogen facilities (Seoul, South Korea) where strand-specific libraries with 250–300 bp insert sizes were built. Samples were sequenced using 150 bp paired-end reads on a Novaseq 6000 platform.

### Raw data quality control and assembly

The quality of the raw reads was assessed for each sample with FastQC v0.11.8 (Andrews 2010). The software Trimmomatic v0.38 (Bolger et al. 2014) was then used to quality-filter the reads and trim the Illumina adapters with the following parameters: LEADING-5 TRAILING-5 SLIDINGWINDOW-5:20 MINLEN-36. Afterwards, random sequencing errors were fixed with the program Rcorrector v1.0.3 Song and Florea 2015).

With no *T. ligericus* genome currently available, we opted to assemble the transcriptome with a de novo approach. Assembly was carried out with Trinity v2.13.2 (Grabherr et al. 2011; Haas et al. 2013) with default parameters. Afterwards, transcript redundancy was removed with Corset v1.0.9 (Davidson and Oshlack 2014). To remove possible contamination, the assembled transcripts were then matched against the NCBI nucleotide database NCBI-nt; (Download; 24/08/2021) and Univec database (Download; 02/04/2019) in Blast-n v2.11.0 (Camacho et al. 2009). Transcripts with less than 100 bp, an e-value of 1e-5, an identity score < 90% and no match to the class Actinopterygii (Taxonomy ID:7898), as well as any transcript that matched against the Univec database were removed from the assembly. The completeness of the transcriptome was assessed both before and after redundancy/contamination removal with BUSCO v3.0.2 (Simão et al. 2015) with lineage-specific libraries of Eukaryota, Metazoa, Vertebrata and Actinopterygii. Transrate v1.0.3 (Smith-Unna et al. 2016) was then used to assess transcriptome integrity before and after redundancy/contamination removal.

### ORF prediction and transcriptome annotation

Open Reading Frames (ORFs) prediction was carried out with Transdecoder v5.3.0 (https://transdecoder.github.io/). The software Blast-p v2.12.0 (Camacho et al. 2009) and hmmscan of hmmer2 package v2.4 (Finn et al. 2011) were used to search for homology and proteins by matching the transcripts against UniProtKB/Swiss-Prot48 (Bateman et al. 2017) and PFAM (Punta et al. 2012) databases. Both TransDecoder output in.gff format and the de novo assembly were processed with Gtf/Gff Analysis Toolkit (AGAT) v0.8.0 (Dainat et al. 2020) to prepare the file.gff3 for functional annotation. Afterwards, AGAT tool was used to standardize file formatting of protein and transcript fasta files. Functional annotation was carried out with InterProScan v5.44.80 (Quevillon et al. 2005) and Blast-n/p/x search. The proteins were mapped against NCBI-RefSeq—Reference Sequence Database (Download; 10/03/2022), NCBI-nr—non-redundant database (Download; 15/12/2021) and InterPro database (Download; 30/03/2019) using Blast-p/x tools implemented in DIAMOND v2.0.13 (Buchfink et al. 2014). The transcripts were searched in nt and nr databases of NCBI using Blast-n and Blast-x tools, respectively, from NCBI and DIAMOND. Finally, the blast outputs and InterProScan outputs were concatenated and integrated into the annotated gff3 file using AGAT. Gene names were assigned following the best blast hit and ranked as follows: 1 for Blast-p Hit from RefSeq database; 2 for Blast-p Hit from NCBI-nr database; 3 for Blast-x Hit from NCBI-nr database; 4 for Blast-n Hit from NCBI-nt database.

### Relative gene expression

Relative gene expression was assessed in each of the five tissues using two distinct methods: the first approach involved utilizing the de novo assembled transcriptome. This analysis was conducted with Trinity scripts, aligning the trimmed and corrected reads against the transcriptome using Bowtie2 v2.3.0 (Langmead and Salzberg 2012) and quantifying gene expression with RSEM v1.2.31 (Li and Dewey 2014). For the second approach, a reference genome-based pipeline was applied, using the available *T. thymallus* genome as a reference. The reads were mapped to the *T. thymallus* genome using HISAT2 v2.2.1 (Kim et al. 2015), and the resulting SAM files were converted to BAM format and sorted using SAMtools v1.9 (Li et al. 2009). StringTie v2.1.2 Pertea et al. 2015) was used to generate read counts for each mapped dataset.

## Results and discussion

### RNA sequencing

Raw reads obtained for each tissue were deposited at the NCBI Sequence Read Archive database under the accession numbers SRR26130661-SRR26130665 and the BioProject PRJNA1019285. The non-redundant transcriptome assembly is provided on NCBI databases under the accession number GKPV00000000. Both redundant and non-redundant transcriptome assemblies (*_trinity_filtered.fasta.gz and *_transcriptome.fasta.gz) are also provided on Figshare platform (10.6084/m9.figshare.24174285), together with transcripts (*_genes_final.fasta.gz), mRNA (*_mrna_final.fasta.gz), ORF prediction (*_cds_final.fasta.gz), proteins ORF prediction (*_proteins_final.fasta.gz), annotation files (*_annotation.gff3.gz) and relative gene expression results.

Considering the conservation status of *T. ligericus*, a vulnerable species with small and fragmented populations, the specimen’s capture and sacrifice must be justified. Therefore, we chose to sequence only one individual of the species. However, the robustness of the results is improved by sequencing and analyzing five different tissues, increasing the number of represented genes. In this case, the use of one individual did not represent a limitation for the generation of a robust transcriptome. Although the use of one sample precludes statistical analyses requiring higher representativeness (e.g., differential gene expression, population genetics), it represents a framework to develop less invasive methods for producing transcriptome resources. This approach has been previously used for other taxa such as bivalves, chondrichthyans and teleosts (Carruthers et al. 2018; Lan et al. 2018; Machado et al. 2018, 2020; Gomes-dos-Santos et al. 2022), providing extremely valuable genomic resources.

### Raw datasets and pre-assembly processing quality control

The sequencing produced 18,708,599 paired-end raw reads for the brain tissue, 22,621,561 for the gill, 23,200,311 for the gonad, 23,250,910 for the liver and 23,118,988 for the muscle (Table 1).

**Table 1:** Number of raw reads obtained from the sequencing of each of the five sampled tissues, i.e., brain, gill, gonad, liver and muscle

	Brain	Gill	Gonad	Liver	Muscle
Raw sequencing reads	18,708,599	22,621,561	23,200,311	23,250,910	23,118,988
Reads after Trimmomatic clean-up	18,285,939	22,130,039	22,718,756	22,742,055	22,682,786

For each tissue, the final number of reads retained per sample before the assembly process (after Trimmomatic removal) is shown

**Fig. 2:**
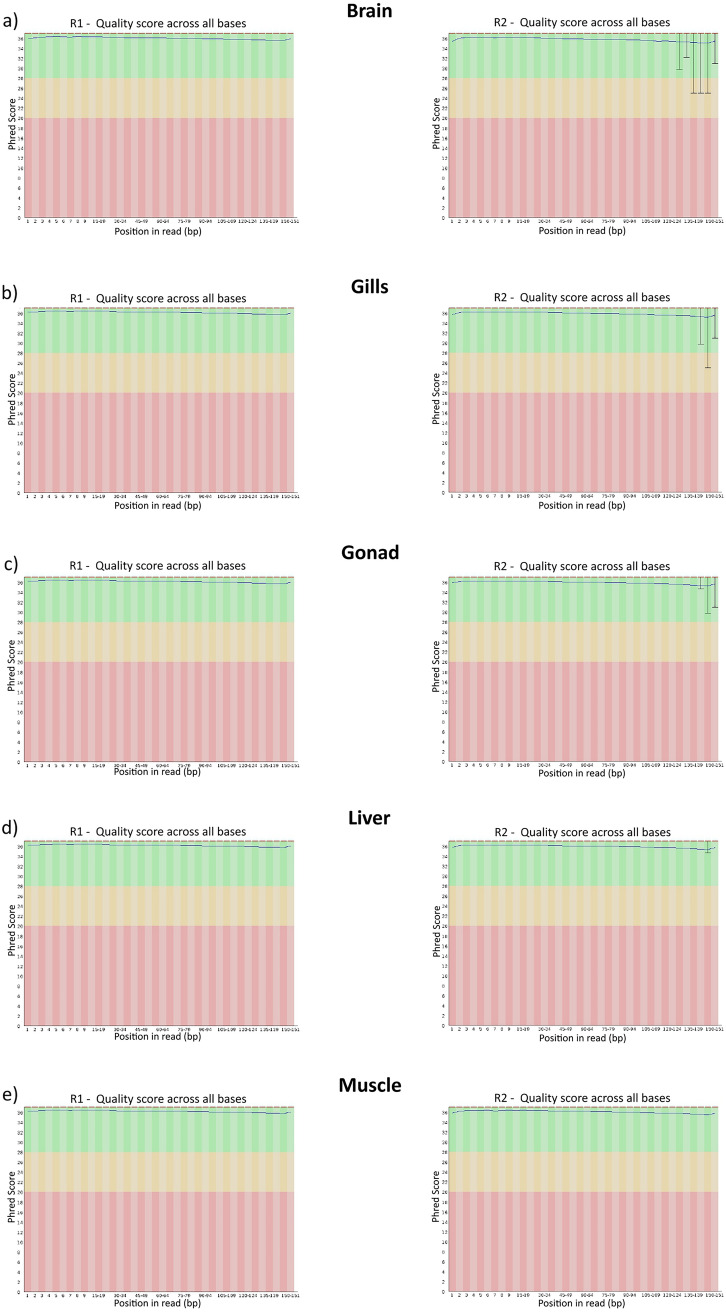
Quality control report of the forward (R1) and reverse (R2) trimmed reads obtained in FastQC for each of the five tissues analyzed. The x-axis indicates each base pair of the reads, whereas the y-axis indicates Phred scores used to measure the DNA quality. Green, orange and red areas represent high, medium and low-quality values, respectively. As shown in the plots, all the reads from each of the five tissues analyzed showed high Phred scores values within the green area

After read trimming and correction (carried out respectively with Trimmomatic and Rcorrector) the quality check performed in fastQC returned high Phred score values (> 20) for both forward and reverse sequences (Fig. 2). The number of reads retained after trimming and used in the assembly are shown in Table 1.

### Transcriptome assembly, annotation and relative gene expression

The newly assembled redundant transcriptome included 1,147,286 sequences and an N50 length of 922 (Table 2). The Benchmarking Universal Single-Copy Orthologs (BUSCO) allowed evaluation of the gene completeness by matching the transcriptome with Eukaryota, Metazoa, Vertebrata and Actinopterygii orthologues database (respectively 303, 978, 2,586 and 4,584). BUSCO scores show a high level of gene completeness with more than 90% (complete + duplicated) genes for the four inferred groups (Euk, Met, Vet, Act) (Table 2).

**Table 2:** Structural and gene content assessment of the redundant and non-redundant transcriptome assemblies (Transrate and BUSCO scores)

Transrate scores											
	Number of transcripts	N bases	Mean transcript length (bp)	Number of transcripts over 1 K nt	Number of transcripts over 10 K nt	N90 transcript length (bp)	N70 transcript length (bp)	N50 transcript length (bp)	N30 transcript length (bp)	N10 transcript length (bp)	Percentage of GC (%)
Redundant	1,147,286	752,159,332	655.58151	177,319	428	282	492	922	1,755	3,620	0.44874
Non-redundant	279,365	280,214,152	1003.03958	90,928	113	509	823	1,221	1,909	3,529	0.44524

Busco analysis (%)				
	Euk (n: 303)	Met (n: 978)	Vet (n: 2586)	Act (n: 4584)
Redundant	T:100;C:99.0%[S:20.8%,D:78.2%], F:1.0%,M:0.0%	T:100;C:99.2%[S:22.1%,D:77.1%], F:0.8%,M:0.0%	T:98.2;C:89.7%[S:24.0%,D:65.7%], F:8.5%,M:1.8%	T:94;C:84.6%[S:23.5%,D:61.1%], F:9.4%,M:6.0%
Non-redundant	T:99.4;C:99.1%[S:74.3%,D:24.8%], F:0.3%,M:0.6%	T:98.2;C:97.4%[S:71.0%,D:26.4%], F:2.0%,M:0.6%	T:97.6;C:88.7%[S:72.2%,D:16.5%], F:8.9%,M:2.4%	T:92.6;C:83.3%[S:65.7%,D:17.6%], F:9.3%,M:7.4%

The redundant transcriptome includes a higher number of duplicated and partial transcripts than the non-redundant

*Euk* Eukaryota; *Met* Metazoa; *Vet* Vertebrata; *Act* Actinopterygii. *T* Total BUSCOs found (complete + fragmented); *C* complete BUSCOs; (single + duplicated); *S* Complete and single-copy BUSCOs; *D* Complete and duplicated BUSCOs; *F* Fragmented BUSCOs; *M* Missing BUSCOs

After the assembly, redundancy removal was carried out with Corset. The program removed about 75% from the initial amount of transcripts increasing the N50 from 922 to 1,221 and causing a general improvement in the quality of the assembly (Table 2). This is also reflected in the BUSCO scores. Although the percentages of matched genes are slightly lower, the percentages of single-copy genes increased significantly in the four classes (Euk, Met, Vet, Act) (Table 2), highlighting the importance and efficiency of redundancy removal. Afterwards, the non-redundant transcriptome was decontaminated with Blast-n searches against the NCBI-nt and Univec databases. Of the 68,242 predicted proteins 54,176 were functionally annotated (Table 3). All the annotation files can be consulted in FigShare (10.6084/m9.figshare.24174285). The present work provides a high-quality transcriptome assembly with an N50 over 1000 bp and BUSCO scores that are congruent with those of recently published high-quality assemblies (e.g., Lan et al. 2018; Machado et al. 2018; Moreno-Santillán et al. 2019; Li et al. 2019; Shen et al. 2020; Gomes-dos-Santos et al. 2022. In our study, BUSCO scores improved remarkably after redundancy removal with the number of duplicated genes decreasing up to 50%. The de novo transcriptome assembly was obtained following a robust pipeline previously applied successfully by our team in fish as well as in other organisms such as freshwater bivalves (Machado et al. 2020; Gomes-dos-Santos et al. 2022).

**Table 3:** General statistics of the structural and functional annotation of the transcriptome

Structural annotation	N transcripts	N cdss	N exons	Tot gene length	Tot cds length	Tot exons length	Mean gene length	Mean cds length	Mean exons length	
	279,442	68,242	68,242	280,286,098	57,054,459	110,810,116	1003	836	1623	–

Functional annotation InterPro/Blast*	CDD	Coils	GO	Gene3D	Hamap	InterPro	KEGG	MetaCyc	MobiDBLite	PIRSF
	15,539	9158	26,321	33,102	384	41,633	1865	1565	27,711	1563

	PRINTS	Pfam	ProSitePatterns	ProSiteProfiles	Reactome	SFLD	SMART	SUPERFAMILY	TIGRFAM	Total
	8,518	37,953	9970	22,964	10,299	91	19,389	31,616	1551	54,176
	Blast-p/x/n hits*									
	46,869									

Additionally, the complete tables of relative gene expression obtained with de novo and referenced pipeline for each of the five tissues are also available in FigShare (10.6084/m9.figshare.24174285). Gene expression quantification was performed both using Fragments Per Kilobase of Transcript Per Million (FKPM) and Transcripts Per Million (TPM) metrics. The gene IDs followed the gff annotation file. The count of genes with TPM and FKPM values > 1, for each tissue, as identified with the two methods, are displayed in Table 4. The de novo approach yielded a significantly higher number of transcripts with TPM and FPKM values > 1 than the approach using genome reference pipeline. This outcome is expected due to the differences underlying the two approaches. The referenced pipeline is based on complete genes, while the de novo pipeline is based on transcripts. Although the first approach is deemed as a more conservative and reliable approach, important information may be lost if the reference genome used belongs to a distinct species, as is the case herein, i.e., the *T. thymallus* genome. In this study, the de novo pipeline that used the *T. ligericus* transcriptome here produced as a reference limited the loss of species-specific information. On the other hand, while working with transcripts through the de novo pipeline it is inevitable to include, for example, different unigene entries for two non-contiguous reads that may belong to the same gene. Considering the different advantages that each of the approaches brings to the analyses, both results are provided.

**Table 4:** Results obtained from the relative gene expression analyses in the de novo and reference methods for the five tissues sampled

	Brain		Gills		Gonad		Liver		Muscle	
	FKPM	TPM	FKPM	TPM	FKPM	TPM	FKPM	TPM	FKPM	TPM
De novo	31.20	32.60	39.17	36.84	38.31	35.46	21.87	22.61	18.85	19.42
Reference	59.60	65.52	53.36	57.85	55.57	60.25	32.89	36.02	35.76	38.97

Quantification of the genes was carried out with two commonly used metrics: Fragments Per Kilobase of transcript per Million (FKPM) and Transcripts Per Million (TPM). Only values of FPKM and TPM > 1 are considered

## Conclusions

This is the first transcriptomic resource of *T. ligericus*, which is an important step forward considering that transcriptome assemblies are only available for a distinct *Thymallus* species, i.e., the European *T. thymallus*. Despite increasing research attention on the genomics of salmonids, whole genome assemblies are only available for a few species (e.g. Berthelot et al. 2014; Christensen et al. 2018a,b; Varadharajan et al. 2018). This is largely due to both the large size (ca. 3 Gb) and complexity of salmonid genomes, which experienced an ancestral event of whole genome duplication about 80 MY. Previous studies have shown that a quarter of the salmonid genome has undergone multiple lineage-specific re-diploidisation events and that part of it is still undergoing re-diploidisation (e.g. Robertson et al. 2017; Gundappa et al. 2022). These characteristics represent well-known hurdles for whole genome assemblies (see Carruthers et al. 2018). Under this scenario, transcriptomic resources may represent the most suitable and cost-effective solution to generate informative data at the whole genome level. By targeting solely transcribed regions, transcriptomes significantly reduce sequencing costs and facilitate the computational demand and accuracy of the assembly process (e.g., Wang et al. 2019). Moreover, transcriptomes provide fast, informative and valuable resources for meeting the demand for genomic tools to study threatened species (e.g., Connon et al. 2018). This is particularly important for understudied species whose conservation status is already at risk, such as many species of the *Thymallus* genus (Weiss et al. 2021). The importance of transcriptome resources as a tool to enhance the ever-developing field of conservation physiology has been previously demonstrated (e.g., Connon et al. 2018) and several works have shown how transcriptome data can be used to refine species management plans (e.g., Connon et al. 2012; Narum and Campbell 2015; Wellband and Heath 2017).

In conclusion, the results reported here constitute a significant advancement in the study of the vulnerable freshwater salmonid *T. ligericus,* a key resource for this distinctive genus, providing a valuable resource for future studies investigating the vulnerability of this group to present and forthcoming threats.

## Data Availability

All software and respective versions applied for the present work are stated in the Methods section, together with the associated parameters. When parameters are not specified, default settings are used. Raw reads are available at the NCBI Sequence Read Archive database (SRR26130661-SRR26130665; BioProject PRJNA1019285. The non-redundant transcriptome assembly is provided on NCBI databases under the accession number GKPV00000000. Redundant and non-redundant transcriptome assemblies (*_trinity_filtered.fasta.gz and *_transcriptome.fasta.gz), transcripts (*_genes_final.fasta.gz), mRNA (*_mrna_final.fasta.gz), ORF prediction (*_cds_final.fasta.gz), proteins ORF prediction (*_proteins_final.fasta.gz), annotation files (*_annotation.gff3.gz) and relative gene expression results are also provided on Figshare platform (10.6084/m9.figshare.24174285).
